# Pentraxin 3 and the TyG Index as Two Novel Markers to Diagnose NAFLD in Children

**DOI:** 10.1155/2021/8833287

**Published:** 2021-03-08

**Authors:** Xiaolin Ye, Jing Li, Hongyu Wang, Jie Wu

**Affiliations:** ^1^Department of Pediatrics, China Medical University affiliated with Shengjing Hospital, Shenyang, 110004 Liaoning, China; ^2^Department of Gastroenterology, Beijing Children's Hospital, Capital Medical University, National Center for Children's Health, 100045 Beijing, China

## Abstract

**Background:**

The diagnosis of NAFLD requires a liver biopsy, which is difficult in children. This study explored the diagnostic value of pentraxin 3 (PTX-3) and the triglyceride-glucose (TyG) index for NAFLD in children.

**Methods:**

Sixty-eight children with NAFLD were selected as study subjects, and 68 healthy children enrolled during the same period served as controls. The TyG index was calculated, serum PTX-3 expression was detected by enzyme-linked immunosorbent assay, and the correlations between PTX-3 or the TyG index and clinical and biochemical indicators were analyzed. A receiver operating characteristics curve analysis and area under the curve (AUC) were used to evaluate diagnostic accuracy.

**Results:**

Serum PTX-3 level and the TyG index of the NAFLD patients were significantly higher than those of the healthy controls (*P* < 0.001), which was closely related with the BMI, ALT, and insulin resistance. The AUC of PTX-3 for diagnosing NAFLD was 0.731 (95% confidence interval [CI] 0.646-0.806), and the AUC of the TyG index for diagnosing NAFLD was 0.765 (95% CI 0.682-0.835). The AUC of PTX-3, the TyG index, and ALT for the combined diagnosis of NAFLD was 0.964 (95% CI 0.916-0.989).

**Conclusion:**

PTX-3 and the TyG index are novel diagnostic biomarkers for NAFLD, as they effectively improved the diagnostic accuracy for NAFLD when combined with ALT.

## 1. Introduction

Nonalcoholic fatty liver disease (NAFLD) is a clinical pathological syndrome with the major feature of hepatocyte fatty degeneration caused by dysmetabolism. NAFLD is regarded as a hepatic manifestation of metabolic syndrome. NAFLD is a reversible process during the early stage and fatty liver status changes with disease progression. About 10–20% of patients with NAFLD progress to nonalcoholic steatohepatitis (NASH), and most eventually progress to portal hypertension, hepatic cirrhosis, and hepatocellular carcinoma [1, 2]. Children with NAFLD have a longer life expectancy and an increased risk of complications. Therefore, the early diagnosis and treatment of NAFLD is of great significance.

NAFLD has no specific clinical manifestations, so diagnosing the disease early is difficult. A liver biopsy is the “gold standard” to diagnose NAFLD, but its disadvantages greatly limit its application in the diagnosis of children, including invasiveness, sampling error, and possible complications (e.g., pain and bleeding at the puncture site and pneumothorax) [3, 4]. The clinically common liver function injury indicators (e.g., serum alanine aminotransferase [ALT] and aspartate transaminase [AST]) do not match the actual severity of hepatocyte injury and fail to accurately reflect the severity of NAFLD in children [5]. Liver ultrasonography, magnetic resonance imaging, computed tomography, and other imaging techniques have limited diagnostic value and cannot be used to make a quantitative diagnosis [6]. Therefore, it is important to identify proper serum markers for early diagnosis, evaluation, and prognosis of NAFLD.

Pentraxin-3 (PTX-3) is a member of the pentraxin super-family and is a classical mediator of inflammation and a marker of the acute-phase reaction. Studies have suggested that PTX-3 is strongly associated with the occurrence of such diseases as type II diabetes, atherosclerosis, and septicemia [7–10]. PTX-3 demonstrates a significant positive correlation with the disease activity index and the severity of fatty degeneration and liver fibrosis in adults with NAFLD [11, 12]. In addition, the triglyceride- (TG-) glucose (TyG) index is calculated using TGs and fasting plasma glucose (FPG). Some studies have shown that the TyG index has a high diagnostic value for NAFLD in adults, and a higher TyG index corresponds to significantly increased morbidity from NAFLD [13, 14]. The present study aimed to evaluate the diagnostic value of the TyG index and PTX-3 for NAFLD in children and to identify novel biomarkers possibly applicable to an early diagnosis of NAFLD in children.

## 2. Methods

### 2.1. Subjects

Sixty-eight children diagnosed with NAFLD by an imaging examination were selected as study subjects and were admitted to the Pediatric Digestive Department, Shengjing Hospital, China Medical University from January 1 to May 31, 2020. Sixty-eight healthy children served as controls and received a health check at the Child Healthcare Department during the same period. This study conformed with the Declaration of Helsinki, and it was implemented strictly following a protocol approved by the Medical Ethics Committee of Shengjing Hospital, China Medical University. All patients and participants were informed of the study and voluntarily signed informed consent.

The inclusion criteria were (1) patients 3–14 years; (2) patients who met the diagnostic criteria for NAFLD among children in the Expert Consensus on Diagnosis and Treatment of Nonalcoholic Fatty Liver Disease in Children formulated jointly by the Chinese Society of Pediatric Endocrinology and Metabolism and the Chinese Society of Pediatric Digestology in 2018 [15]; and 3) patients whose legal guardians signed informed consent for study participation, or patients ≥10 years who signed informed consent for study participation together with their legal guardians.

The exclusion criteria were (1) patients with other liver diseases (e.g., viral hepatitis, autoimmune hepatitis, or Wilson's disease); (2) patients with other diseases possibly causing an increase of PTX-3 (e.g., chronic inflammatory disease, heart failure, autoimmune disease, and rheumatoid disorders); (3) patients who recently used drugs that could affect serum transaminase levels; (4) patients with recent medical conditions that could possibly affect serum transaminase and PTX-3 levels (e.g., infection); (5) patients with a history of chromosomal disease or inherited metabolic disease; (6) patients who required total parenteral nutrition or other support, possibly inducing a fatty liver; and (7) patients who refused the relevant examinations or who had incomplete clinical data.

### 2.2. Collection and Treatment of Blood Samples

Venous blood samples were collected into vacuum tubes during the early morning from subjects in a fasting state. The samples were immediately sent to the Laboratory Department of Shengjing Hospital, China Medical University, for testing the relevant indicators. The samples were centrifuged at 1,000 rpm for 15 min in a high-speed centrifuge within 30 min of collection, and the plasma was separated and extracted into EP tubes. The plasma samples were numbered and stored at −80°C to detect PTX-3 levels by enzyme-linked immunosorbent assay (ELISA).

### 2.3. Measurement of Clinical Data and Biochemical Analysis of the Blood Samples

Clinical data (including height and body mass index [BMI]) were collected. After collecting the fasting blood samples, the following biochemical indicators were determined using an automatic biochemical analyzer: fasting blood glucose (FBG), total cholesterol (TC), triglycerides (TGs), high-density lipoprotein cholesterol (HDL-C), low-density lipoprotein cholesterol (LDL-C), small and dense LDL cholesterol, apolipoprotein A1, apolipoprotein B, fasting insulin (FINS), and uric acid (UA). Plasma PTX-3 levels were measured by ELISA.

The BMI, homeostatic model assessment of insulin resistance (HOMA-IR), and the TyG index were calculated using the following formulas:

(1) $$\begin{array}{c} \begin{array}{c} \text{HOMA}‐\text{IR}=\text{FPG}\left(\text{mmol}/\text{L}\right)\times \text{FINS}\left(\text{mU}/1\right)/22.5\\ \text{TyG}=\text{Ln}\left[\text{TG}\left(\text{mg}/\text{dl}\right)\times \text{FPG}\left(\text{mg}/\text{dl}\right)/2\right]\\ \text{BMI}=\text{body weight}\left(\text{kg}\right)/\text{body heigh}{\text{t}}^{2}{\left(\text{m}\right)}^{2}. \end{array} \end{array}$$

### 2.4. Statistical Methods

The SPSS21.0 statistical software (SPSS Inc., Chicago, IL, USA) was used for data collection and analysis, and the Graphpad Prism 8 software (GraphPad Software Inc., La Jolla, CA, USA) was employed for the graphics. Qualitative data are presented as numbers (*n*) and percentages (%) and were compared with the *x*^2^ test. Normal distributed quantitative data are expressed as mean ± standard deviation (SD) and were compared using the independent sample *t*-test. Abnormally distributed data are presented as medians and interquartile range and were compared with the Mann–Whitney *U*-test. The correlations between two variables were analyzed by Pearson's correlation or Spearman's correlation analyses. Logistic regression was performed with NAFLD as the dependent variable and ALT, PTX-3, and the TyG index as independent variables, and the confounding factors were age and sex. The diagnostic value of single and combined marker scores for NAFLD was evaluated using a receiver operating characteristic (ROC) curve. The optimal cut-offs of various parameters were determined by calculating Youden's index, and their sensitivity, specificity, and area under curve (AUC) for diagnosing NAFLD were computed. The AUC was compared with the Z test. A *P* value < 0.05 was considered significant.

## 3. Results

### 3.1. General Subject Data

There were 43 males and 21 females (age: 10 [9, 11] years) in the NAFLD group and 32 males and 32 females (age: 10 [7, 12] years) in the healthy control group (Table 1). No significant differences in age or sex were observed between the groups (*P* > 0.05). The BMI, ALT, AST, *γ*-glutamyl transpeptidase (GGT), FINS, TG, HDL-C, LDL-C, UA, and HOMA-IR were significantly higher in the NAFLD group than those in the healthy control group (*P* < 0.05), but no significant differences were observed in FPG, TC, ApoA1, or ApoB between the two groups (*P* > 0.05).

### 3.2. The Expression of PTX-3 and the TyG Index in the Healthy Control and NAFLD Groups

**Figure 1:**
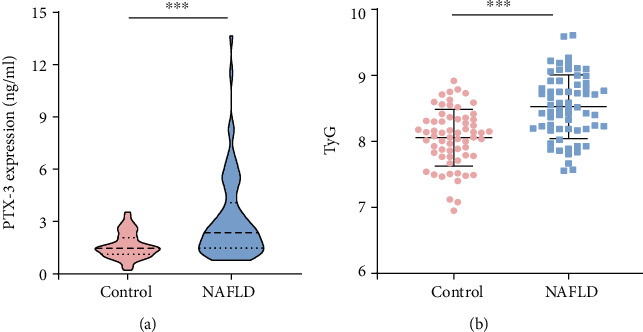
The expression of PTX-3 and the TyG index. (a) PTX-3 expression level detected by ELISA. (b) TyG index. ^∗∗∗^, compared with the healthy control group, *P* < 0.001.

The median PTX-3 expression level and the TyG index were 1.57 ng/ml and 8.06 in the healthy control group and 2.52 ng/ml and 8.53 in the NAFLD group. Serum PTX-3 level and TyG index increased significantly (*P* < 0.001) in the NAFLD group compared with the healthy control group (Figure 1).

### 3.3. Correlations between PTX-3 or the TyG Index and the Clinical Biochemical Indicators

The correlation analysis between serum PTX-3 level or the TyG index and the clinical biochemical indicators of the NAFLD patients is shown in Table 2. PTX-3 was significantly positively correlated with BMI, ALT, AST, GGT, ApoB, FINS, UA, and HOMA-IR, while the TyG index demonstrated significant positive correlations with BMI, ALT, AST, GGT, ApoB, FBG, FINS, TC, LDL-C, UA, and HOMA-IR.

### 3.4. Diagnostic Efficacy of PTX-3 and the TyG Index for NAFLD

**Figure 2:**
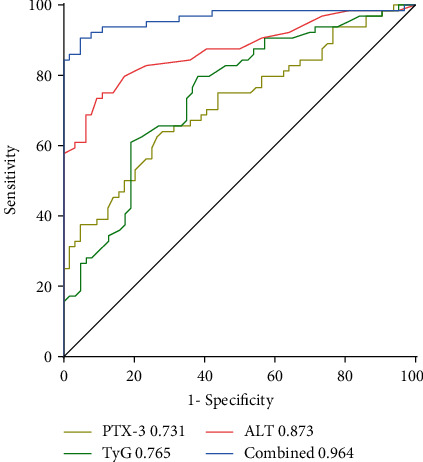
ROC curves of PTX-3, the TyG index, and ALT. The combination of PTX-3, the TyG index, and ALT had the largest AUC (0.964), followed by ALT (AUC: 0.873), the TyG index (AUC: 0.765), and PTX-3 (AUC: 0.731).

The diagnostic efficacy of ALT, PTX-3, and the TyG index for NAFLD, as well as their combination, was analyzed using a ROC curve (Figure 2). Sensitivity, specificity, AUC, and the optimal cut-off of ALT for diagnosing NAFLD were 75%, 89.06%, 0.873, and 20 U/l, while those of PTX-3 were 65.62%, 71.87%, 0.731, and 1.89 ng/ml, and those of the TyG index were 81.25%, 60.94%, 0.765, and 8.16. These results show that ALT had a higher diagnostic value than PTX-3 and the TyG index in children with NAFLD (*P* < 0.05). After adjusting for age and sex, the combined score of these three variables was obtained by logistic regression, and the combined prediction was analyzed using a ROC curve. The results revealed that sensitivity, specificity, and AUC of the combination of ALT, PTX-3, and the TyG index for diagnosing NAFLD were 90.62%, 95.31%, and 0.964, and the negative predictive value and positive predictive value increased gradually; such a combination demonstrated significantly improved diagnostic efficacy (*P* < 0.01) compared with that of ALT alone (Table 3).

## 4. Discussion

This study demonstrated that PTX-3 and the TyG index are novel diagnostic biomarkers for NAFLD in children. The serum PTX-3 level and the TyG index increased significantly and were positively correlated with BMI, the transaminases, and HOMA-IR, and they effectively improved the diagnostic efficacy for NAFLD when combined with ALT.

The pathogenesis of NAFLD has not been fully clarified, but it is believed that NAFLD is closely associated with lipid dysmetabolism, insulin resistance (IR), oxidative stress, and the host inflammatory reaction. Similar to obesity, chronic inflammation and IR are considered the central link in the development of NAFLD, and lipid accumulation is the major feature [1, 16, 17]. Chronic inflammation may induce a massive loss of hepatocytes and hence exacerbate the severity of various hepatic conditions, including NAFLD [18], type I NKT, and neutrophil extracellular traps that seem to have a role in the regulation of chronic inflammation [19, 20]. In clinical practice, ALT is usually used to evaluate the severity of liver injury in NAFLD patients. ALT is recommended as a conventional screening method for NAFLD in several NAFLD diagnostic and treatment guidelines and has been used as a marker for oxidative stress and the inflammatory reaction [2, 21, 22]. However, applying ALT for diagnosing NAFLD is controversial. One study showed that 79% of patients with NAFLD diagnosed by liver ultrasonography have a normal serum ALT level [23]; our study results revealed that serum ALT level increased (≥40 U/L) in 59.3% of NAFLD patients. These findings indicate that a higher ALT level cannot be used to accurately identify patients with NAFLD; thus, biomarkers more sensitive than ALT are needed.

PTX-3 is a soluble pattern-recognition receptor that plays a critical role in the innate immune response and is an important marker for the permanent inflammatory reaction [24–26]. Some studies have suggested that the serum PTX-3 expression level is low in the healthy population but increases significantly in patients with inflammation and infectious disease [27–29]. In addition, PTX-3 is important for diagnosing liver disease. According to some studies, PTX-3 can be used as an inflammatory marker for the prognosis of patients with hepatic cirrhosis [30]; serum PTX-3 expression markedly increases in adult NASH patients; thus, PTX-3 can be used as a biomarker for the differential diagnosis of NASH and help evaluate the severity of liver fibrosis in patients with NASH [12, 31]. However, other studies have concluded that PTX-3 has no value in the diagnosis of NAFLD or the differential diagnosis of NASH [32]. A recent study showed that serum PTX-3 expression level is significantly higher in patients with a type II diabetes comorbidity and NAFLD than that in diabetic patients, and PTX-3 is positively correlated with TC, LDL-C, and TGs. However, few studies have been conducted on the diagnostic value of PTX-3 for NAFLD in children. Our study results demonstrate that the serum PTX-3 levels in NAFLD patients increased compared with those in the healthy controls and were positively correlated with the transaminases, UA, HOMA-IR, and the TyG index, which is consistent with a previous report [33]; PTX-3 was not correlated with TC or TGs, which is different from study results of adult NAFLD. The possible reasons include the following: (1) our study subjects were children, of which fewer cases had hypercholesterolemia and hypertriglyceridemia, compared with adult patients; (2) only 68 NAFLD patients were included in this study, so the small sample size may have affected the results.

The TyG index was first proposed to evaluate IR by Simental-Mendia et al., and it is helpful to diagnose fatty liver degeneration. Some studies have revealed that the TyG index is related with the progression of diabetes, cardiovascular diseases, and NAFLD [13, 34, 35]. Based on our results, the TyG index was evidently higher in children with NAFLD than in healthy children and was positively correlated with BMI, TC, LDL-C, UA, and HOMA-IR, indicating that the TyG index may be useful to evaluate blood lipid metabolism and IR levels in NAFLD children, which is coincident with a previous report [36]. Our study results revealed positive correlations between the TyG index and ALT, AST, GGT, and UA levels, suggesting that the TyG index may reflect hepatocyte injury and purine metabolism in children with NAFLD. In accordance with these findings, it is reasonable to use the TyG index, calculated with TG and FPG, as an effective diagnostic tool for NAFLD.

In this study, we compared the diagnostic value of PTX-3, the TyG index, and ALT for NAFLD. The results showed that the sensitivity, specificity, and optimal cut-off of ALT for diagnosing NAFLD were 75%, 89.06%, and 20 U/l. The TyG index had higher sensitivity and lower specificity (81.25% and 60.94%) and an optimal cut-off of 8.16 compared with ALT, while PTX-3 had lower sensitivity and specificity (65.62% and 71.87%) and an optimal cut-off of 1.89 ng/ml. These findings suggest that the diagnostic value of PTX-3 and the TyG index for NAFLD cannot be a substitute for ALT. However, the combination of PTX-3, the TyG index, and ALT demonstrated a significant increase in diagnostic sensitivity and specificity (90.62% and 95.31%) and had an AUC of 0.964.

This study had a small sample size, and no liver biopsies were performed to accurately evaluate the severity of liver injury in NAFLD patients, so there may be some limitations. This was a cross-sectional study, and the effects of the TyG index and PTX-3 on the prognosis of NAFLD patients were not observed further. Therefore, subsequent work should enlarge the sample size and identify some novel indicators applicable to comprehensively evaluate the severity of NAFLD and the general status of patients to provide new clues for the early diagnosis and prognosis of NAFLD in children.

In conclusion, this study demonstrated that the combination of PTX-3, the TyG index, and ALT significantly increased the diagnostic rate of NAFLD, and the optimal cut-offs of PTX-3 and the TyG index for diagnosing NAFLD were 1.89 ng/ml and 8.16. Our findings are of great significance considering the invasiveness of liver biopsy and that the application of imaging examinations in children is limited to a certain extent; thus, serum markers are still the first choice for screening NAFLD. PTX-3 and the TyG index can be used as noninvasive biomarkers to assist in the diagnosis of NAFLD in children.

## Figures and Tables

**Table 1:** General subject data.

Variable	NAFLD patients	Healthy controls	*P* value
	(*n* = 64)	(*n* = 64)	
Age (years)	10 (9, 11)	10 (7, 12)	0.205^b^
Male (%)	43 (67.2)	32 (50.0)	0.072^c^
Overweight and obese *n* (%)	62 (96.9)	19 (29.6)	<0.001^c^
BMI (kg/m^2^)	25.8 (23.4, 28.3)	17.8 (17.1, 18.4)	<0.001^b^
BMI z-score	0.71 (0.26, 1.20)	-0.80 (-0.94, -0.69)	<0.001^b^
ALT (U/l)	61.0 (19.5, 145.0)	13.5 (10.0, 16.0)	<0.001^b^
AST (U/L)	31.0 (19.3, 65.0)	23.0 (18.0, 25.8)	<0.001^b^
GGT (U/L)	33.5 (19.0, 63.3)	12.0 (9.3, 14.8)	<0.001^b^
FPG (mmol/l)	5.0 (4.7, 5.2)	4.9 (4.3, 5.2)	0.115^b^
Fasting insulin (mU/l)	16.3 (11.3, 26.6)	9.7 (7.2, 12.3)	<0.001^b^
TC (mmol/l)	4.2 (3.5, 4.7)	4.2 (3.8, 4.6)	0.989^b^
TG (mmol/l)	1.2 (1.0, 1.8)	0.8 (0.7, 1.1)	<0.001^b^
HDL-C (mmol/l)	1.1 (1.0, 1.2)	1.3 (0.9, 1.6)	0.031^b^
LDL-C (mmol/l)	2.6 (2.0, 3.0)	2.3 (1.9, 2.7)	0.046^b^
ApoA1 (g/l)	1.30 ± 0.22	1.31 ± 0.34	0.757^a^
ApoB (g/l)	0.8 (0.6, 1.0)	0.7 (0.6, 0.8)	0.061^b^
Uria acid (*μ*mol/l)	371.5 (336.0, 468.5)	289.0 (266.0, 315.8)	<0.001^b^
PTX-3 (ng/ml)	2.52 (1.60, 4.48)	1.57 (1.19, 2.12)	<0.001^b^
HOMA-IR	3.6 (2.2, 5.5)	1.0 (0.7, 1.7)	<0.001^b^
TyG	8.53 ± 0.48	8.06 ± 0.43	<0.001^a^

a = Student's *t*-test; b = Mann–Whitney *U*-test; c = Pearson's chi-square test. Data are expressed as mean ± SD, median (25th–75th interquartile range), or number of cases (%), as appropriate. BMI: body mass index; AST: aspartate aminotransferase; ALT: alanine aminotransferase; GGT: *γ*-glutamyltransferase; FPG: fasting plasma glucose; TC: total cholesterol; TG: triglycerides; HDL-C: high-density lipoprotein cholesterol; LDL-C: low density lipoprotein cholesterol; HOMA-IR: homeostatic model assessment of insulin resistance; ApoA1: Apolipoprotein A1; ApoB: apolipoprotein B; PTX-3: pentraxin-3; TyG: triglyceride-glucose.

**Table 2:** Correlations between PTX-3 or the TyG index and the clinical biochemical indicators.

	PTX3		TyG	
	*r*	*P* value	*r*	*P* value
BMI (kg/m^2^)	0.365	<0.001	0.408	<0.001
ALT (U/l)	0.4	<0.001	0.388	<0.001
AST (U/L)	0.194	0.029	0.228	0.01
GGT (U/L)	0.31	<0.001	0.493	<0.001
FPG (mmol/l)	0.091	0.307	0.308	<0.001
Fasting insulin (mU/l)	0.327	<0.001	0.379	<0.001
TC (mmol/l)	0.006	0.948	0.214	0.016
TG (mmol/l)	0.169	0.056	0.974	<0.001
HDL-C (mmol/l)	-0.047	0.598	-0.064	0.47
LDL-C (mmol/l)	0.14	0.114	0.354	<0.001
ApoA1 (g/l)	0.01	0.91	0.119	0.181
ApoB (g/l)	0.174	0.05	0.336	<0.001
Uria acid (*μ*mol/l)	0.274	0.002	0.372	<0.001
HOMA-IR	0.343	<0.001	0.407	<0.001

**Table 3:** Comparison of the predictive value for diagnosing NAFLD between PTX-3, the TyG index, or ALT and their combination.

	Cut-off value	Sensitivity (95% CI)	Specificity (95% CI)	AUC (95% CI)	PPV (%)	NPV (%)
ALT	20 U/l	75 (62.6-85.0)	89.06 (78.8-95.5)	0.873 (0.802-0.925)	0.75	0.89
PTX-3	1.89 ng/ml	65.62 (52.7-77.1)	71.87 (59.2-82.4)	0.731 (0.646-0.806)^∗∗^	0.66	0.72
TyG	8.16	81.25 (69.5-89.9)	60.94 (47.9-72.9)	0.765 (0.682-0.835)^∗^	0.81	0.61
Combined model		90.62 (80.7-96.5)	95.31 (86.9-99.0)	0.964 (0.916-0.989)^∗∗^	0.91	0.95

Note: ^∗^, compared with the AUC of ALT, *P* < 0.05; ^∗∗^, compared with the AUC of ALT, *P* < 0.01. AUC: the area under the ROC curve; PPV: positive predictive value; NPV: negative predictive value.

## Data Availability

The data used to support the findings of this study are available from the corresponding author upon request.
